# Cooperation between cGAS and RIG-I sensing pathways enables improved innate recognition of HIV-1 by myeloid dendritic cells in elite controllers

**DOI:** 10.3389/fimmu.2022.1017164

**Published:** 2022-12-07

**Authors:** Enrique Martin-Gayo, Ce Gao, Marta Calvet-Mirabent, Zhengyu Ouyang, Mathias Lichterfeld, Xu G. Yu

**Affiliations:** ^1^ Ragon Institute of Massachusetts General Hospital (MGH), Massachusetts Institute of Technology (MIT) and Harvard, Massachusetts General Hospital, Cambridge, MA, United States; ^2^ Universidad Autónoma de Madrid, Immunology Unit, Hospital Universitario de la Princesa, Madrid, Spain; ^3^ Infectious Disease Divisions, Brigham and Women’s Hospital and Massachusetts General Hospital, Boston, MA, United States

**Keywords:** HIV-1, innate immunity, myeloid dendritic cells, DNA sensor, RNA sensor

## Abstract

**Introduction:**

Spontaneous control of HIV-1 replication in the absence of anti-retroviral therapy (ART) naturally occurs in a small proportion of HIV-1-infected individuals known as elite controllers (EC), likely as a result of improved innate and adaptive immune mechanisms. Previous studies suggest that enhanced cytosolic immune recognition of HIV-1 reverse transcripts in conventional dendritic cells (mDC) from EC enables effective induction of antiviral effector T cell responses. However, the specific molecular circuits responsible for such improved innate recognition of HIV-1 in mDC from these individuals remain unknown.

**Results and methods:**

Here, we identified a subpopulation of EC whose mDC displayed higher baseline abilities to respond to intracellular HIV-1 dsDNA stimulation. A computational analysis of transcriptional signatures from such high responder EC, combined with functional studies, suggested cytosolic recognition of HIV-1 dsDNA by cGAS, combined with sensing of viral mRNA by RIG-I after polymerase III-mediated HIV-1 DNA transcription.

**Discussion:**

Together, our work identifies collaborative networks of innate sensing pathways that enhance cell-intrinsic abilities of mDC to induce antiviral innate responses against HIV-1; these observations might be useful for the therapeutic induction of effective antiviral immune responses.

## Introduction

More than a decade ago, a rare group of HIV-1 infected individuals who were able to spontaneously control HIV-1 replication in the absence of antiretroviral therapy (ART) was identified (1–4). These HIV-1 controllers consist of a heterogeneous group including individuals maintaining low-level, drug-free viremia (<2000 copies/ml), some of whom eventually lose control and require ART initiation. An even smaller subset of controllers, frequently termed “elite controllers”, is able to maintain undetectable levels of HIV-1 replication for decades, in the absence of antiretroviral therapy. Recent studies suggest that natural control of HIV-1 is associated with integration of intact proviruses in heterochromatin regions, likely as a result of effective cellular immune responses that successfully eliminated intact proviruses integrated in more accessible chromatin locations (5). The type, etiology and evolution of such powerful immune responses, however, remains an area of active investigation. HIV-1-specific T cells are currently regarded as the main backbone of antiviral immunity in EC, and their critical role for natural control is supported by a large body of data. Interestingly, recent work from our group suggested increased abilities of primary myeloid dendritic cells (mDC) from EC to sense HIV-1 through cytosolic immune recognition pathways (6), leading to the acquisition of a CD64^Hi^ PD-L1^Hi^ mDC phenotype functionally characterized by improved abilities to expand and stimulate polyfunctional HIV-1-specific T cells (7). These previous studies demonstrated that innate HIV-1 sensing in mDC from EC was dependent on the presence of cytoplasmic HIV-1 reverse transcripts, the signal transducer TBK1 (8), and the intracellular DNA sensor cGAS. However, precise mechanisms underlying effective innate HIV-1 immune recognition in mDC from EC remain insufficiently understood. In this study, we show that cell-intrinsic sensing of HIV-1 in mDC from EC can involve viral RNA and DNA replication products. In particular, we identified Polymerase III as a molecular bridge capable of mediating transcription of HIV-1 dsDNA into mRNA and therefore, providing a substrate for RNA-sensing pathways. Moreover, we confirmed that the RNA-sensor RIG-I together with cGAS were major players cooperatively mediating the detection of HIV-1 in mDC from EC.

## Results

### A subgroup of ECs with improved cell-intrinsic recognition of HIV-1 dsDNA

**Figure 1:**
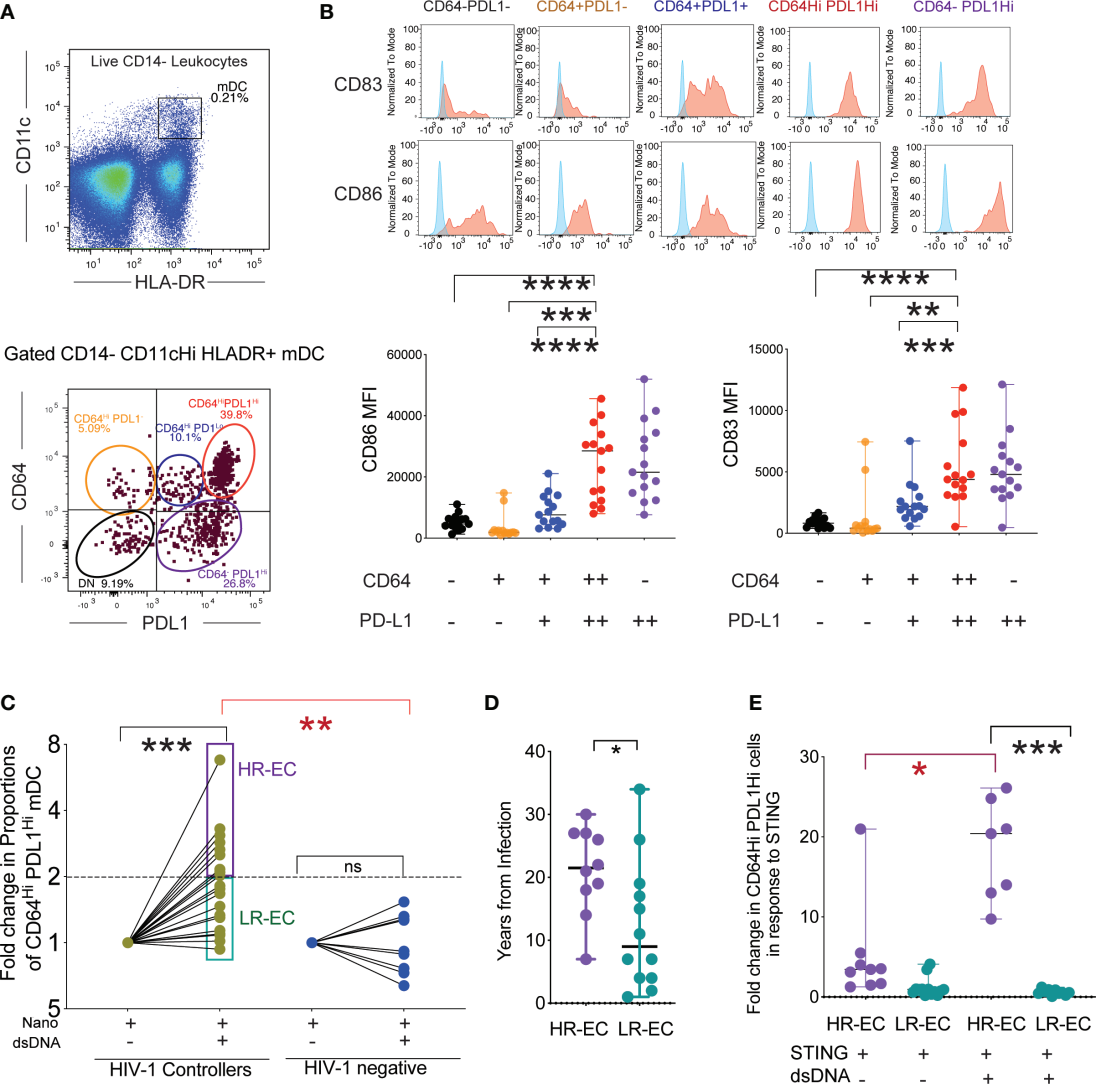
Identification of a subgroup of EC with high mDC response to intracellular HIV -1 dsDNA stimulation. **(A)** (upper diagram) Representative Flow cytometry gating strategy defining mDC as CD14^-^ HLADR^+^ CD11c^Hi^ cells within PBMC stimulated with HIV-1 Gag dsDNA. (lower diagram): Identification of different subpopulations of mDC, based on surface expression levels of CD64 and PD-L1 following stimulation with nanoparticles loaded with HIV-1 Gag dsDNA. **(B)** (upper panel): Representative flow cytometry histograms reflecting expression of activation markers CD86 and CD83 in subsets of mDC defined by expression signatures of CD64 and PD-L1. Background FMO controls (shown in blue) for each population are overlayed on each histogram. (lower panel) Mean Fluorescence Intensity (MFI) of CD86 and CD83 in indicated mDC subsets after stimulation with nanoparticles loaded with HIV-1 Gag dsDNA. Data from n=15 EC participants are shown. (**p<0.01, ***p<0.001, ****p<0.0001, two-tailed Wilcoxon test). **(C)** Fold change in proportions of CD64^Hi^ PD-L1^Hi^ mDC from EC and HIV-1 negative donors in response to stimulation with nanoparticles loaded with HIV-1 Gag dsDNA compared to nanoparticles alone (Nano). High responder (HR-EC; purple) and low responder (LR-EC; light green) EC subpopulations are defined. Data are represented in a Log2 scale. Statistical significance of differences among EC or compared to HIV-1 negative donors were calculated using two-tailed matched-pairs Wilcoxon test (black; *** p<0.001) or Mann Whitney tests (red; **p<0.01), respectively. **(D)** Years since infection in HR-EC and LR-EC subgroups. (*p<0.05, Mann Whitney test). **(E)** Fold change in proportions of CD64^Hi^ PD-L1^Hi^ mDC from HR-EC (violet) and LR-EC (green) after stimulation with soluble 2´3´-c-di-AM(PS) (STING agonist) alone or in combination with HIV-1 Gag dsDNA. Statistical significance of differences within and among EC subgroups were calculated using two-tailed matched pairs Wilcoxon tests (black) or Mann Whitney U tests (red), respectively, *p<0.05; ***p<0.001.

In our previous work, we demonstrated that following exposure to HIV-1, mDC from EC develop a CD64^Hi^ PD-L1^Hi^ phenotype, paired with improved antigen-presenting properties (7); however, the precise mechanisms underlying this process are not clear. To address this, we first determined whether maturation of CD14^-^ CD11c^+^ HLA-DR^+^ mDC into cells defined as CD64^Hi^ PD-L1^Hi^ (**Figure 1A**) could also be induced upon stimulation with nanoparticles loaded with HIV-1-Gag dsDNA probes. This approach allowed the intracellular delivery of HIV-1 dsDNA in PBMC cultures in the absence of other viral components (see methods) and the subsequent flow cytometry analysis of phenotypic changes in mDC into CD64^Hi^ PD-L1^Hi^ or other cell populations with different levels of these markers that results from cell-intrinsic immune recognition (**Figure 1A**). Using this *in vitro* system, we observed that exposure of mDC from EC to HIV-1 dsDNA nanoparticles recapitulated the previously observed evolution of an CD64^Hi^ PD-L1^Hi^ phenotype (**Figures 1B, C**). These CD64^Hi^ PD-L1^Hi^ cells and CD64^-^ PDL1^Hi^ cells expressed the highest levels of CD83 (**Figure 1B**). In addition, CD86 tended to be higher in the CD64^-^ PDL1^Hi^ followed by CD64^Hi^ PD-L1^Hi^ cells and compared with cells expressing either CD64 alone or intermediate levels or negative expression for the two markers (**Figure 1B**). We then quantified proportions of activated CD64^Hi^ PD-L1^Hi^ mDCs from EC and healthy donors after stimulation with HIV-1 dsDNA nanoparticles. As shown in **Figure 1C** and **Supplemental Figure 1A**, consistent with our previous studies, mDC from EC generally displayed a higher proportion of CD64^Hi^ PD-L1^Hi^ mDC compared to cells from HIV-1 negative donors, suggesting elevated abilities in mDC from EC to recognize HIV-1. Notably, within the study cohort of EC, phenotypic changes in response to viral immune recognition varied profoundly: One group, termed “high responder EC” (HR-EC), defined by the ability to increase populations of CD64^Hi^ PD-L1^Hi^ mDC in response to dsDNA by a cutoff of at least two fold compared to baseline, while in a different group of EC, termed “low responder EC” (LR-EC), innate immune recognition did not visibly differ from a control cohort of HIV-1 negative persons (**Figure 1C**, **Supplemental Figure 1A**). Notably, proportions of mDC displaying lower expression of CD64 and PD-L1 after stimulation with HIV-1 dsDNA were not significantly different between HR-EC and LR-EC (**Supplemental Figure 1B**), but proportions of immature CD64^-^ PD-L1^-^ mDC were higher in LR-EC (**Supplemental Figure 1B**).

While both HR-EC and LR-EC have normal CD4+ T cell counts in blood (**Supplemental Figure 1C**), HR-EC showed a more limited number of viral blips, and had been infected with HIV-1 for a significantly longer time, suggesting that HR-EC represent a more definitive example of superior control of HIV-1 infection (**Figure 1D**). Compatible with this view, higher proportions of non-protective and high-risk HLA-B alleles (B*35:01; B*39:10; B*50:01; B*702; B*801) were found in LR-EC, while proportions of protective HLA-B alleles (B*44:02; B*57:01; B*57:03; B*27:05; B*13:02; B*14:02; B*51:01) were slightly higher in HR-EC (60%) compared to LR-EC (42%, p=0.02) (**Supplementary Figure 1D**). Together, these studies demonstrate that a subgroup of EC displays improved cell-intrinsic immune responses to HIV-1 dsDNA.

### Transcriptional signatures in mDC from HR-EC suggest activation of both DNA and RNA HIV-1 sensing pathways

Innate immune recognition of HIV-1 in myeloid cells can be mediated by cGAS (6). Binding of cGAS to dsDNA allosterically activates its catalytic activity and leads to the production of 2′3′ cyclic GMP–AMP (cGAMP), a second messenger molecule and potent agonist of Stimulator of Interferon Genes (STING) (9), which triggers phosphorylation of IRF3 *via* TBK1, allowing for subsequent nuclear entry of IRF3 (10). To discriminate whether the previously observed improved recognition of HIV-1 dsDNA in HR-EC, was either exclusively due to the activation of the cGAS/STING sensing pathways or involved additional underlying mechanisms, we compared the phenotypic profile of mDC following exposure to saturating concentrations of the STING agonist 2´3´-di-AM (PS) alone, or in combination with HIV-1 dsDNA-loaded nanoparticles. As shown in **Figure 1E**, while HR-EC tended to respond better to STING stimulation (relative to LR-EC), but the induction of CD64^Hi^ PD-L1^Hi^ cells was significantly higher in response to HIV-1 dsDNA-nanoparticles. In contrast, mDC from LR-EC displayed increased proportions of CD64^-^ PD-L1^-^ cells after exposure to HIV-1 dsDNA-nanoparticles (**Supplemental Figure 1E**). These data suggest that more complex HIV-1 DNA sensing pathways are involved in the induction of CD64^Hi^ PD-L1^Hi^ in mDC from HR-EC.

**Figure 2:**
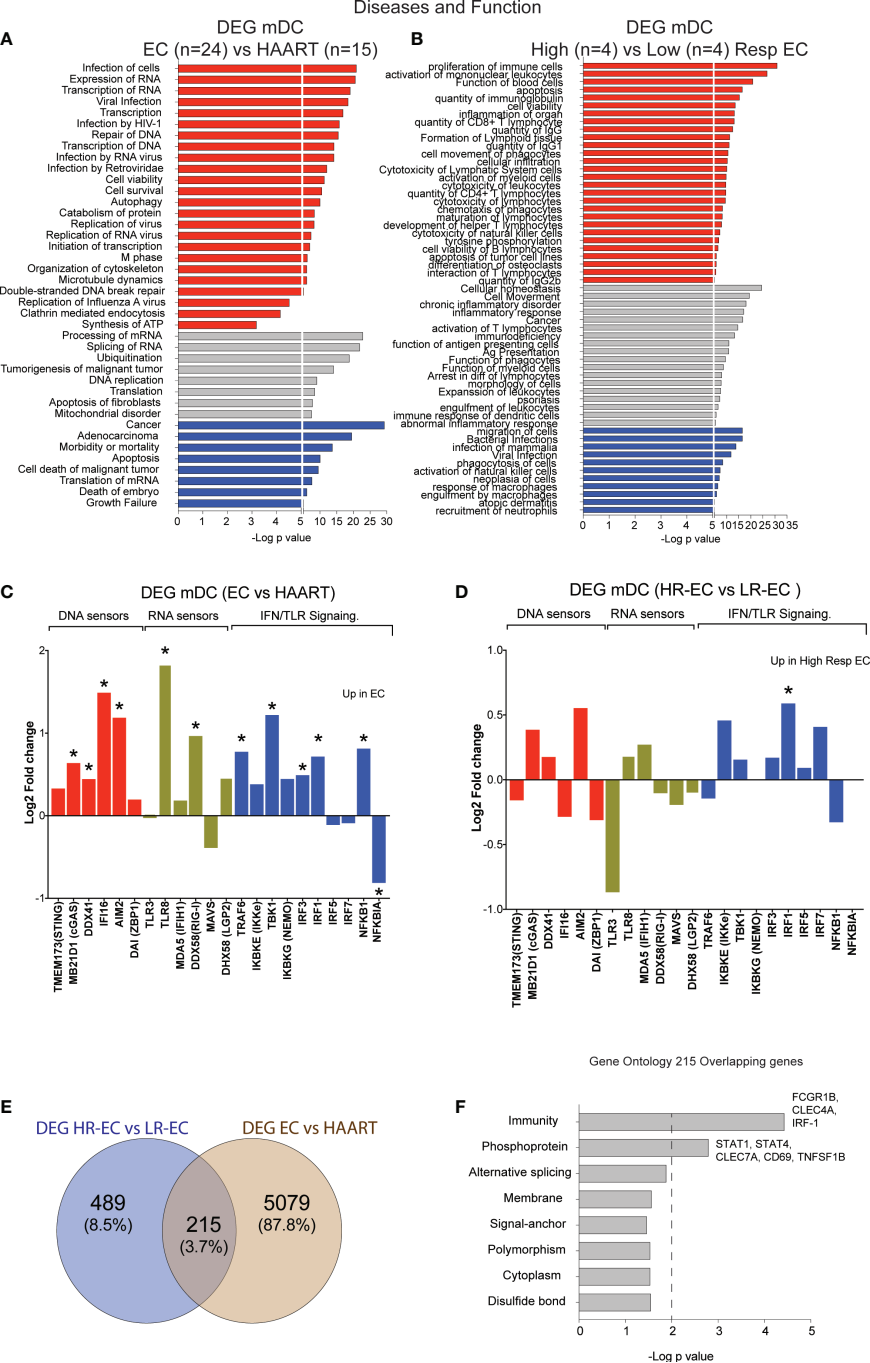
Identification of transcriptional signatures of mDC from total EC and from High vs Low EC responders. **(A, B)** Ingenuity Pathway Analysis (IPA) for selected diseases and functions for DEG between mDC from total EC (n=24) vs ART-treated individuals (n=15) **(A)** and High-Responder (HR, n=4) vs Low-Responder (LR, n=4) EC subgroups **(B)**. Pathways predicted to be upregulated (red), downregulated (blue) or with non-directional predictions (grey) are highlighted. **(C, D)** Log2 Fold change in expression of selected DNA sensors, RNA sensors and IFN pathway elements included in the list of DEG for EC vs HAART-treated individuals **(C)** and for HR vs LR EC **(D)**. Statistically significant (FDR-adjusted p<0.05 EC vs HAART; nominal p<0.05 HR-EC vs LR-EC) DEGs are highlighted with a star. **(E)** Venn diagram representing overlap between DEG from total EC vs HAART participants and DEG from HR-EC vs LR-EC subgroups. **(F)** Biological pathways enriched within the 215 overlapping genes defined in **(E)**, as determined by DAVID gene ontology analysis. *p < 0.05.

To evaluate molecular pathways that may be involved in the detection of HIV-1 dsDNA in mDC from HR-EC, we performed RNA-seq in *ex vivo* isolated, unstimulated primary cells. Firstly, we compared transcriptional signatures in mDC from a cohort of EC (n=24) versus mDC from (n=15) HIV-1 progressors receiving antiretroviral therapy (ART). Samples used for the transcriptional analysis included n=8 HR-EC and n=8 LR-EC based on their ability to respond to *in vitro* stimulation with dsHIV-1 DNA, allowing for a discrimination of their respective transcriptional signatures. Ingenuity pathway analysis (IPA) of differentially expressed genes (DEG) from mDC from EC compared to ART-treated persons (**Supplemental Table 1**) suggested the upregulation of biological processes associated with both DNA and RNA sensing, viral infection and replication of HIV-1 or RNA viruses, as well as transcription and expression of RNA (**Figure 2A**), suggesting improved innate detection of viral nucleic acids in mDC from EC. In addition, DEG distinguishing mDC from HR-EC compared to cells from LR-EC (**Supplemental Table 2**) were functionally involved in activation of antiviral immunity, cytotoxicity, cell migration, antigen presentation and inflammation (**Figure 2B**), compatible with higher levels of functional activation in these cells. A more targeted analysis of mRNA expression levels of intracellular microbial DNA- and RNA-sensors and their downstream signaling molecules suggested that, compared to mDCs from ART-treated patients, cells from EC expressed significantly higher levels of the microbial DNA sensors cGAS, IFI16 and AIM2, in addition to higher levels of the RNA sensors TLR8 and RIG-I (**Figure 2C**). However, expression of these sensors was not significantly different between HR-EC versus LR-EC, suggesting that mRNA expression levels of these molecules did not account for different mDC phenotypes evolving in response to HIV-1 dsDNA stimulation in the two groups of EC (**Figure 2D**). Interestingly, IRF1 mRNA, encoding for a molecule involved in type I IFN signaling and in the RIG-I-MAVS pathway (11), was significantly upregulated both in HR-EC and in total EC compared to LR-EC and ART-treated individuals, respectively (**Figures 2C, D**). Moreover, DEG from mDC in HR-EC compared to LR-EC and ART-treated persons also involved differential expression of mRNAs of STAT1 and STAT4, which act as downstream mediators of type I IFN signaling (**Figures 2E, F**).

**Figure 3:**
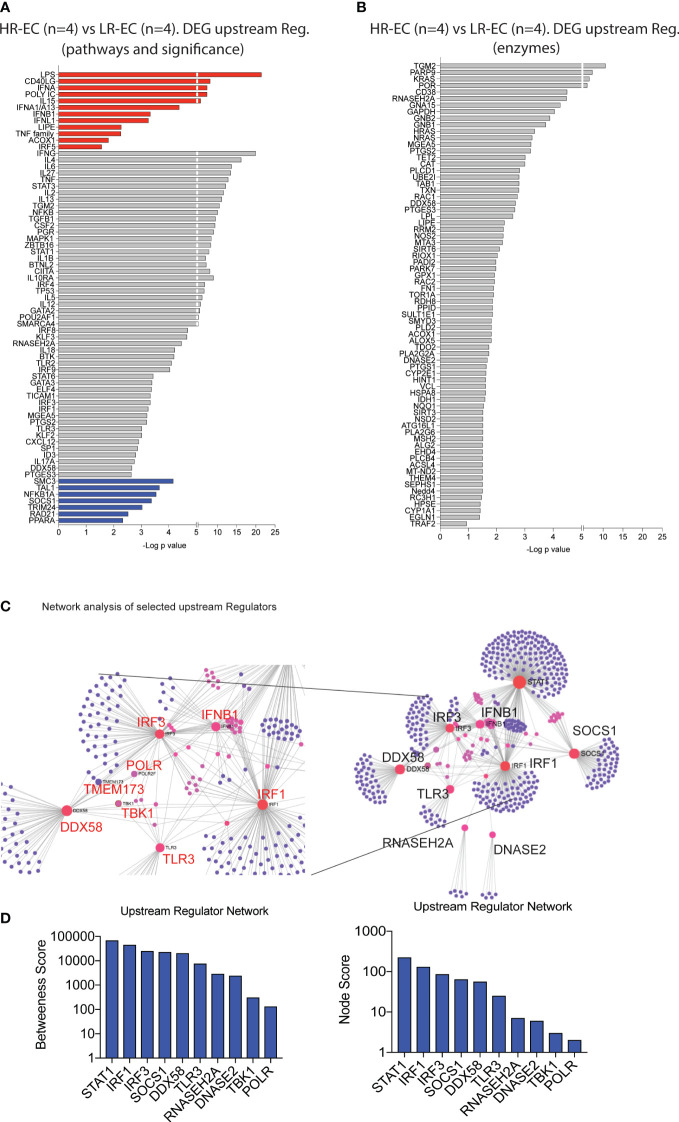
Identification of potential upstream regulators for the high responder EC. **(A, B)** Selected upstream regulators of DEG distinguishing high responder (HR-EC) vs low responder (LR-EC) EC subgroups, as determined by Ingenuity Pathway Analysis. Data reflect all immune pathways (left) or only molecules with enzymatic activity (right). Molecules with a predicted positive upstream effect are highlighted in red, while molecules with a predicted negative effect are in blue. **(C)** Network interaction of selected upstream regulators identified in **(A)** (right panel) highlighting relevant genes within interaction nodes (red) and connecting genes (pink) as well as individual gene targets (violet). Zooms on the central Gene Nodes are displayed in the left panel. **(D)** Analysis of Betweenness and Node scores of upstream regulator genes included within network defined in **(C)**.

We next identified upstream molecules significantly predicted the specific transcriptional signatures of mDC from HR-EC compared to LR-EC. Interestingly, type I (IFNA, IFNB) and III (IFNL1) IFNs as well as the TLR3/MDA5/RIG-I ligand Poly I:C were the top immune-related molecules predicted to govern the transcriptional signature of mDC from HR-EC (**Figure 3A**); In addition, LPS and the TNF family, were also predicted to be upregulated in cells from HR individuals. In contrast, expression of SOCS1, a gene involved in suppression of type I interferon and cytokine signaling, seemed to be negatively associated with the transcriptional patterns of HR-EC (**Figure 3A**). Notably, RIG-I (DDX58), STAT1 and 3, MAPK, NFKB and TLR-associated proinflammatory cytokines such as IL6, TNF, IL1B were also predicted to participate in upstream regulation of mDC from HR-EC in an unbiased or enzyme-directed analysis (**Figures 3A, B**). Interestingly, enzymes predicted to contribute to upstream regulation of transcriptional signatures in HR-EC mDC also included RNAase H and DNAase II (**Figure 3B**), consistent with the hypothesis that molecules able to restrict microbial RNA and DNA substrates are regulating the mDC gene expression profiles in HR-EC. Therefore, type I IFN and TLR signatures may be active in mDCs from HR. Finally, to better understand the relationship between the upstream regulator candidates identified, we performed a network analysis (12) (**Figure 3C**). As expected, these studies supported the assumption that RIG-I (DDX58) was interconnected and interacted directly or indirectly with known components of the IFN signaling pathways such as STAT1, TMEM173, IRF1, IRF3 and that the potential regulators RNASEGH2A, DNASE2 and SOCS1 were also connected to the network, although they did not directly interact with these genes (**Figures 3C, D**). TBK1 was also part of this network although it did not directly interact with all the mentioned ISGs and regulatory DEGs (**Figures 3C, D**). Importantly, this analysis also identified RNA-polymerase as a molecule interacting between the STING-TBK1-RIG-I pathways (**Figure 3D**). Interestingly, RNA II polymerase 2F was initially identified in the network analysis and we observed significant transcriptional upregulation of many units of RNA polymerase I, II ans III in mDCs from EC compared to HAART progressors (**Supplemental Figure 2A**).

Moreover, several units of RNA pol I and III tended to be upregulated in mDCs from HR-EC compared to LR-EC (**Supplemental Figure 2A**). Interestingly, RNA III polymerase has been shown to mediate RNA synthesis from cytoplasmic DNA during viral infections, leading to RIG-I activation (13). These data, generated in *ex vivo* isolated mDC from EC, suggest that enhanced detection of HIV-1 dsDNA in mDC from HR-EC might involve collaborative interactions between multiple microbial RNA and DNA sensing pathways that culminate in improved induction of cell-intrinsic viral immune recognition.

### RNA Polymerase III transcription of HIV-1 dsDNA permits cooperative immune recognition by RIG-I and cGAS

**Figure 4:**
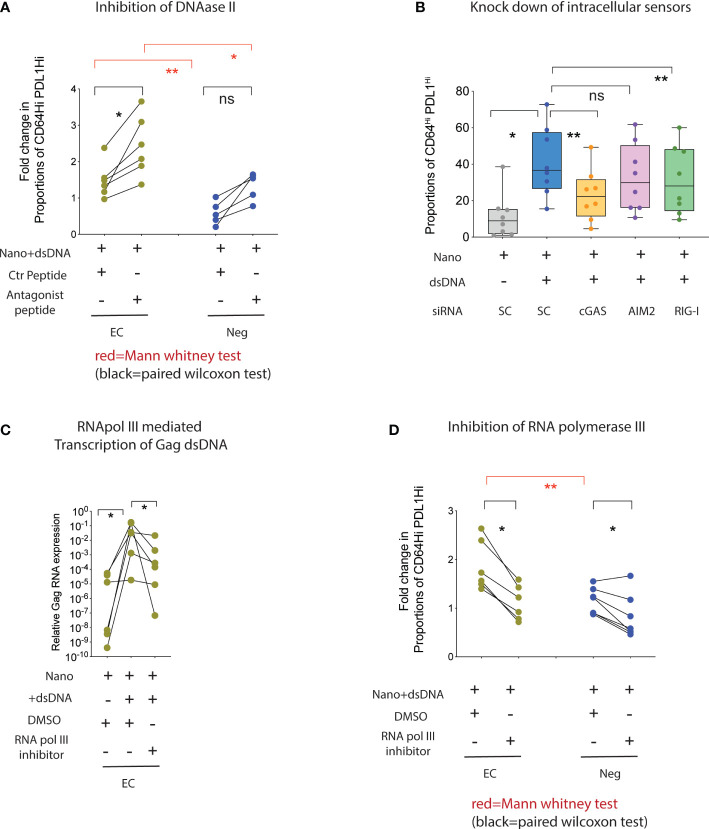
Collaboration of RNA polymerase III, cGAS and RIG-I enhance the detection of intracellular HIV-1 Gag dsDNA in mDC from high responder EC. **(A, D)** Proportions of CD64^Hi^ PD-L1^Hi^ mDC from HR-EC (green) and HIV-1 negative individuals (Neg, blue) after stimulation with nanoparticles loaded with HIV-1 Gag dsDNA or control nanoparticles. Experiments were performed in the presence of either a control peptide or an antagonistic peptide against DNAase II **(A)** or with DMSO or an inhibitor for RNA polymerase III **(D)**. Statistical significance was tested using two-tailed matched pairs Wilcoxon tests (black; * p<0.05) or Mann Whitney tests (red; *p<0.05; **p<0.01), respectively. **(B)** Fold change in proportions of CD64^Hi^ PD-L1^Hi^ mDC in response to stimulation with HIV-1 dsDNA after nucleofection with siRNAs specific for genes encoding the indicated intracellular nucleic acid sensors. Data were normalized to mDC nucleofected with control scramble (SC) siRNAs in the presence of HIV-1 dsDNA stimulation. Baseline normalized levels of mDC nucleofected with scramble siRNA in the absence of HIV-1 dsDNA are also included. **(C)** RT-qPCR analysis of HIV-1 Gag mRNA in mDC from high responder EC cultured with control nanoparticles (Nano) or nanoparticles loaded with HIV-1 gag dsDNA. Experiments were performed in the presence of DMSO or an inhibitor for RNA polymerase III. ns, not significant.

We next stimulated mDC from HR-EC or from healthy donors with HIV-1 dsDNA in the presence or absence of chemical inhibitors or siRNAs directed to some of the upstream regulator candidate molecules previously identified. As shown in **Figure 4A**, inhibition of DNAase II significantly increased the induction of CD64^Hi^ PD-L1^Hi^ cells in mDC from HR-EC, and to a lesser extent in healthy donors, confirming that enhanced innate immune responses in mDC from HR-EC was dependent and associated with the presence of cytoplasmic HIV-1 dsDNA. In contrast, inhibition of SOCS1 did not have a significant effect on the ability of mDC from HR-EC to respond to HIV-1 dsDNA (**Supplemental Figure 2B**). Additional experiments with peptides inhibiting TRIF, a signaling molecule downstream of TLR3, also did not appear to significantly affect the response to dsDNA in mDC from HR-EC either (**Supplemental Figure 2C**). In contrast, pharmacological inhibition of TBK1 and siRNA-mediated gene knockdown of upstream cGAS and RIG-I sensors demonstrated that both molecules significantly contributed to innate immune recognition of HIV-1 dsDNA in mDC (**Supplemental Figures 2C, D**, **Figure 4B**) and MDDC (**Supplemental Figure 2D**), while other intracellular DNA sensors, such as AIM2, did not seem to be involved (**Figure 4B**, **Supplemental Figure 2D**). Thus, our data suggest that intracellular HIV-1 dsDNA can trigger the activation of both DNA and RNA sensing pathways in mDC. Since previous studies have identified RNA III polymerase as a molecule capable of mediating transcription of intracellular DNA feeding the RIG-I-based RNA sensing pathway (13), we asked whether HIV-1 dsDNA can be transcribed by RNA polymerase III, thus producing a substrate for the cell-intrinsic microbial RNA recognition machinery in mDC. As shown in **Figure 4C**, we observed a significant increase in the detection of HIV-1 Gag transcripts in total RNA isolated from mDC from HR-EC incubated with HIV-1 dsDNA; these transcripts were significantly reduced when cells were preincubated with inhibitors for RNA polymerase III. Moreover, acquisition of the CD64^Hi^ PD-L1^Hi^ surface phenotype in mDC from both HR-EC and healthy donors following stimulation with HIV-1 dsDNA was also significantly abrogated in the presence of RNA polymerase III inhibitors (**Figure 4D**).

## Discussion

Our study identified a subgroup of EC whose mDC display superior intrinsic abilities to mount innate responses after stimulation with intracellular HIV-1 dsDNA. This group of HR-EC is characterized by parameters of improved viral control, suggesting that they could represent individuals with most definitive signs of natural HIV-1 immune control. These findings are supported by recent studies that suggest that HIV controllers are indeed a quite heterogeneous population of people living with HIV-1 (5, 14, 15) in whom different mechanisms of immune control might occur. Such EC have been suggested to be “functionally cured”, due to selective elimination of cells harboring proviruses in accessible chromatin, leaving only intact proviruses integrated in gene deserts (5). In addition, our data support previous observations suggesting a superior capacity of mDC from EC to potently induce innate responses against HIV-1 and acquire effective antigen presenting properties associated with viral control of HIV-1 (6, 7, 14). While the accumulation of viral reverse transcripts was previously proposed as one potential cause of innate sensing in mDC from EC, additional viral components such as the viral capsid could also contribute to intrinsic sensing of HIV-1 by mDC (16, 17), although this remains controversial (18). In our study, we tried to minimize these variables by directly incubating mDC with HIV-1 gag dsDNA sequences intracellularly delivered by nanoparticles. Thus, our studies focused on studying molecular mechanisms directly linked to detection of HIV-1 dsDNA. However, this approach may not fully recapitulate the exposure of viral DNA in the cytoplasm in cDC during physiological infection with HIV-1 and may represent a limitation from our study. Moreover, the use of nanoparticles may lead to a higher basal activation state in mDCs and may also influence subsequent responses to viral dsDNA. In addition, we cannot rule out additional HIV-1 immune recognition substrates and viral entry pathways including complement receptor virus internalization (19) inducing RIG-I and TBK-1 and potentially involved in immune recognition of HIV-1. Therefore, additional future studies should investigate the relationship between a larger number of host and viral factors affecting concentrations and detection of cytoplasmic viral nucleic acids in mDC from HR-EC.

An additional important aspect from our work is our ability to link transcriptional signatures present in mDC from EC with enhanced recognition of intracellular HIV-1 dsDNA. In this regard, this analysis allowed us to identify the crosstalk between cGAS and RIG-I pathways and their involvement and interaction with different components of the type I IFN transcriptional signatures of activated mDC from HR-EC individuals. Our previous studies had identified TBK1 as an upstream regulator required for the induction of type I IFN responses in CD64^Hi^ PD-L1^Hi^ mDC (7). In the present study, we demonstrated that the expression of both cGAS and RIG-I, upstream of TBK1, was required for the improved innate recognition of HIV-1 dsDNA in HR-EC. While we were forced to use monocyte derived DCs to simultaneously address the contribution of multiple sensors in the detection of HIV-1 dsDNA, these observations should also be confirmed on primary mDCs. While the role of the cGAS and STING pathways in detecting intracellular dsDNA and HIV-1 reserve transcripts is well recognized (17, 20), RIG-I has received more limited attention as an immune sensor of HIV-1 (21, 22). Although some data suggest that RIG-1 can be involved in detection of HIV-1 during infection (23–25), possible connections between RIG-I-dependent immune recognition and viral DNA sensing pathways have never been studied. Our approach allowed us to identify RNA Polymerase III as a key actor mediating the transcription of transfected cytoplasmic dsDNA into RNA, which may suggest a mechanism by which that reverse transcripts present in the cytoplasm and not completely protected by viral capsid could also be amplified by this enzyme prior to integrating into the genome, allowing for additional innate immune recognition of HIV-1 products in mDC. Interestingly, a cooperative interconnection between the RIG-I pathways and nucleic acid immune recognition by the cGAS/STING (26) or the DNA sensor IFI16 (27) has been suggested before in alternative contexts; and RNA polymerase III-mediated generation of RNA immune recognition substrates has been described before as a means to enhance innate responses against cytoplasmic nucleic acids (13, 28). However, our study extends our understanding on more complex molecular mechanisms other than only cGAS that contribute to enhanced antimicrobial recognition of HIV-1 in mDC from HR-EC. Although other families of RNA polymerases such as RNA polymerase II were also observed upregulated in mDCs from EC and tended to be upregulated in HR-EC, we did not directly study their potential implication in the immune recognition of intracellular dsDNA. In addition, our study suggests that IRF1 could be a biomarker for HR-EC individuals. IRF1 is a molecule originally identified as a regulator of type I IFNs and critical for antiviral immune control, but it has also recently been involved in multiple inflammatory innate immune responses including IFN-independent pathways (29, 30) that might favor HIV-1 replication (31, 32). Therefore, the role of this molecule in the innate sensing of HIV-1 should be investigated in more detail. Despite these limitations, we extend these findings and suggest an intrinsic involvement of cooperative cGAS/STING- and RIG-I-dependent immune recognition in natural immune control of HIV-1. While our data provide additional information about the complex molecular networks that could facilitate innate detection of HIV-1 in at least a subset of HIV-1 controllers, the described immune recognition machinery is also available in cells from the general population. Therefore, alterations in some components of RNA polymerase III, cGAS and RIG-I might represent therapeutically actionable pathways for enhancing innate and HIV-1-specific immune induction against HIV-1. In fact, our work suggest that adjuvants and vaccines designed to target upregulated innate sensors in HR-EC could be helpful to recapitulate the controller phenotype in the general population. In line with this possibility, recent studies suggest that mDCs treated with adjuvants targeting TBK1 are associated with reduced depletion of CD4^+^ T cells and polyfunctional HIV-1 specific CD8^+^ T cells in humanized mice after HIV-1 infection (33). Together, our data further characterize molecular circuits involved in effective sensing of HIV-1 and could be useful for the development of future targeted therapeutic vaccine strategies against HIV-1.

## Methods

### Study participants

For this study we recruited a cohort of n=22 HIV-1 elite controllers (EC) who had maintained < 2000 copies/mL HIV-1 viral load (VL; 20–825 copies/mL, median 103.3 copies/mL) and living with HIV-1 for a median of 16.27 years (range = 1–34 years) in the absence of antiretroviral therapy (CD4+ T cell counts: 297–1786 cells/mL, median 920 cells/mL; n = 8 persons). The EC cohort was subdivided in two groups of high responder (HR-EC, n=10) and low responder (LR-EC; n=11) individuals based on *in vitro* ability to become activated in the presence of viral HIV-1 Gag dsDNA. Their clinical data are summarized here: HIV-1 viral load (VL HR-EC; 20–75 copies/mL, median 20 copies/mL; VL LR-EC; 20–825 copies/mL, median 70.50 copies/mL) and living with HIV-1 for a median of 21.50 (HR-EC; 7–30 years) and 9 years (LR-EC; 1–34 years) and with a median CD4+ T cell counts of 900.5 (HR-EC, 297-1786 cells/ml) and 782 (LR-EC: 497–1543 cells/mL) cells/mL.

For comparison purposes, HIV-1 negative (n = 9) individuals were also recruited for this study. All individuals gave written informed consent; the Institutional Review Board of Massachusetts General Hospital/Partners Healthcare approved the study protocol.

### Isolation of primary conventional dendritic cells and generation of MDDC

Primary circulating CD11c+ mDC were directly purified by negative immunomagnetic selection using the human CD1c (BDCA-1+) Dendritic cell Isolation Kit (miltenyi Biotec) from PBMC of HIV-1 EC and healthy donors. Monocyte-derived-DC (MDDC) were generated from HIV-1 negative circulating adherent monocytes and cultured during 6 days in the presence of recombinant GMCSF and IL-4 as previously described (34).

### *In vitro* stimulation of mDC with HIV-1 Gag dsDNA

Primary mDC from EC (n=22) and healthy individuals (n=9) were exposed to polymeric nanoparticles (Trans IT X2, Myrus Bio) alone or loaded with HIV-1 Gag dsDNA probes generated from complimentary sequences to HIV-1 RNA (forward primer 5´-ATAGTATGGGTAAGCAGGG -3´; reverse primer 5´-CCAATATTTGTCTACAGCC-3´) and the dsDNA was generated by hybridization of the primers Gag-forward and Gag-reverse ssDNA sequences at equimolar concentrations following a protocol previously described; followed by phenotypical and transcriptional analyses using flow cytometry and unbiased RNA-Seq. In some experiments, antagonistic peptides for 2μg/ml DNAase II (SLRLLQWFLWAC) (35), SOCS1 (pJAK2 (1001–1013): LPQDKE[pY]YKVKE; BIO-SYNTHESIS INC) (36), 27μM RNA III Polymerase Inhibitor (CAS 577784-91-9, Calbiochem) or siRNA-mediated gene silencing were used to study the involvement of distinct innate immune recognition pathways in the activation present in mDCs after 16h of exposure to dsDNA. For comparison purposes, in some assays mDCs were also stimulated with 1μg/ml of 2´3´-c-di-AM(PS) STING agonist (*In vivo*Gen).

### Inhibition of TLR during ex vivo Infection of mDC with HIV-1 pseudoviruses

In some experiments, mDC from EC were infected with VSV-G-pseudotyped HIV-1 viruses (MOI=2.4) for 16h as previously described (6) in the absence or the presence of 2μg/mL or 5μg/mL a commercial antagonistic peptide (PEP-0149) preventing TRIF activation, which corresponds to 14 aminoacids near TRIF C-terminus end (Invitrogen; Fisher scientific).

### siRNA mediated gene knockdown in MDDC

1x10^6^ MDDC were nucleofected using 2.5 μM of siRNAs specific for cGAS (MB21D1; L-015607-02-0050), RIG-I (DDX58; Catalog ID:L-012511-00-0050) and AIM2 (Catalog ID:L-012511-00-0050) (Dharmacon ON-TARGETplus siRNA) using the primary P3 lymphocyte buffer and the CM120 protocol in a Lonza´s 4D-Nucleofector system following the manufacturer’s instructions. As a negative control, some MDDC were nucleofected with irrelevant scramble siRNAs. Efficacy of specific siRNA mediated gene knockdown was evaluated by analyzing mRNA levels of target genes compared to cells treated with scramble siRNA.

### RNA-seq and computational analysis of transcriptional signatures of mDC

Total RNA was extracted from sorted Lin- HLADR+ CD11c+ mDC from the blood of a larger cohort of n=24 EC (85% male, 15% female) characterized by a median plasma viral load of 48 copies/ml (range 20-250 copies/ml) and living with HIV-1 for a median of 17.5 years (range = 3–27 years) and with CD4^+^ T cell counts median of 821 cells/mL (range: 407-1684 cells/mL) and n=15 HIV-1 positive individuals on antiretroviral therapy (HAART) with undetectable viremia (<20 copies/mL) and diagnosed a median of 11 years (min-max; 1.27 years) and with a median CD4^+^ T cell counts of 909/mL (min-max; 398-1367) patients using the Qiagen RNeasy Micro Kit. Subsequently, RNA-Seq libraries from mDC were generated as previously described (37). Briefly, SMART-seq2 (37) was used to prepare whole transcriptome amplification (WTA) and tagmentation-based libraries, and samples were sequenced on a NextSeq 500 Instrument (Illumina). Subsequently, sequences were aligned using the Hg38 human genome database by Bowtie 2 (38), and transcripts per million (TPM) values were obtained for each sample by RNA-Seq using Expectation-Maximization (RSEM) (39). TPM values were then normalized among all samples using the upper quantile normalization method. For a first set of analyses mDC RNA-seq data from all EC and all HAART individuals was compared. In subsequent analyses, RNA-seq data from a total of n=8 EC with known high (n=4) or low (n=4) *in vitro* response to HIV-1 dsDNA stimulation in mDC were compared. Pathway analyses were performed using Ingenuity Pathway Analysis and DAVID software. Additional gene network images were obtained from selected upstream regulator lists using the NetworkAnalyst software (12).

### Quantification of HIV-1 Gag mRNA by RT-qPCR

Total mRNA was extracted from mDC cultured for 24h in the presence of nanoparticles alone or nanoparticles loaded with HIV-1 Gag dsDNA probes using *mir*Vana™ Isolation Kit (Life Technologies™). Subsequently, cDNA was generated and analysis by RT-qPCR of HIV-1 Gag transcripts were quantified using specific Primers, as previously described (6).

### Statistics

Differences were tested for statistical significance using a two-tailed Mann Whitney U or Wilcoxon matched pairs tests, respectively. Statistical significance was corrected for multiple comparisons, when appropriate, using a Kruskal Wallis test and Dunn’s *post-hoc* test.

## Data availability statement

The data presented in the study are deposited in the GEO repository, accession number GSE218587.

## Ethics statement

The studies involving human participants were reviewed and approved by Massachusetts General Brigham. The patients/participants provided their written informed consent to participate in this study.

## Author contributions

EM-G, XY and ML designed and supervised the study and prepared the manuscript. EM-G performed most of the experiments. MC-M contributed to siRNA-mediated silencing experiments. CG, ZO performed computational and biostatistical analysis. All authors contributed to the article and approved the submitted version.

## Supplementary material

Supplementary Figure 1 Impact of stimulation with HIV-1 Gag dsDNA and STING agonists on proportions of mDC subpopulations. **(A)** Proportions of CD64^Hi^ PD-L1^Hi^ mDC from EC and HIV-1 negative donors in response to stimulation with nanoparticles loaded with HIV-1 Gag dsDNA compared to nanoparticles alone (Nano). High responder (HR-EC; purple) and low responder (LR-EC; light green) EC subpopulations are defined. Statistical significance of differences among EC or compared to HIV-1 negative donors were calculated using two-tailed matched-pairs Wilcoxon test (black; *** p<0.001) or Mann Whitney tests (red; **p<0.01), respectively. **(B)** Fold change in proportions of CD64^Hi^ PD-L1^int^, CD64^Hi^ PD-L1^-^, CD64 PD-L1^+^ and CD64^-^ PD-L1^-^ mDC subsets from HR-EC (violet) and LR-EC (green) after stimulation with HIV-1 Gag dsDNA stimulation. **(C)** Peripheral blood CD4^+^ T cell counts, HIV-1 plasma viral loads (VL) in HR-EC and LR-EC subgroups. (*p<0.05, Mann Whitney test). Empty circles on VL plot represent viremia below limit of detection. **(D)** Pie Charts representing distribution of study participants expressing either only protective (green), only high risk (orange) or either both (blue) or neither (grey) protective and high-risk HLA-B alleles on the HR-EC and LR-EC subgroups. Statistical significance was calculated using a Chi-square test. **(E)** Fold changes in proportions of mDC subsets in HR-EC (violet) and LR-EC (green) after stimulation with HIV-1 Gag dsDNA in the presence of soluble 2´3´-c-di-AM(PS) (STING agonist). Statistical significance of differences within and among EC subgroups were calculated using two-tailed matched pairs Wilcoxon tests (black) or Mann Whitney U tests (red), respectively, *p<0.05; ***p<0.001.

Supplementary Figure 2 Inhibition and siRNA-mediated gene knockdown of potential upstream regulators in mDC from HR-EC. **(A)** Log2 fold-change of transcriptional expression of members of three different HIV RNA polimerases in mDC from either EC compared to individuals on HAART (left) or high responder ECs (HR-EC) compared to low responder ECs (LR-EC; right). **(B)** Fold change in proportions of CD64^Hi^ PD-L1^Hi^ mDC after stimulation with nanoparticles loaded with HIV-1 Gag dsDNA in EC (green) and HIV-1 negative individuals (blue) in the presence of a SOCS1-specific inhibitor. Corresponding controls with DMSO and irrelevant peptide are also shown. **(C)** Representative flow cytometry dot plots showing expression of CD64 vs PD-L1 in mDC form n=4 HR-EC following exposure to VSV-G-pseudotyped HIV-1 virus in the presence or absence of the indicated concentration of the TRIF inhibitory peptides preventing TLR3 signaling and 1uM of the Bx795 TBK-1 inhibitor. Proportions of CD64^Hi^ PD-L1^Hi^ cells are gated. **(D)** RT-qPCR analysis of transcriptional levels of indicated intracellular nucleic acid sensors after nucleofection of MDDC with scramble or specific siRNAs against each of the target genes. Statistical significance was calculated using a two-tailed matched-pairs Wilcoxon test. *p<0.05; **p<0.01.

## References

[1] Chen H Li C Huang J Cung T Seiss K Beamon J. CD4+ T cells from elite controllers resist HIV-1 infection by selective upregulation of p21. J Clin Invest (2011) 121(4):1549–60. doi: 10.1172/JCI44539 PMC3069774 21403397

[2] Migueles SA Laborico AC Shupert WL Sabbaghian MS Rabin R Hallahan CW. HIV-Specific CD8+ T cell proliferation is coupled to perforin expression and is maintained in nonprogressors. Nat Immunol (2002) 3(11):1061–8. doi: 10.1038/ni845 12368910

[3] Saez-Cirion A Pancino G. HIV Controllers: a genetically determined or inducible phenotype? Immunol Rev (2013) 254(1):281–94. doi: 10.1111/imr.12076 23772626

[4] Walker BD Yu XG. Unravelling the mechanisms of durable control of HIV-1. Nat Rev Immunol (2013) 13(7):487–98. doi: 10.1038/nri3478 23797064

[5] Jiang C Lian X Gao C Sun X Einkauf KB Chevalier JM. Distinct viral reservoirs in individuals with spontaneous control of HIV-1. Nature (2020) 585(7824):261–7. doi: 10.1038/s41586-020-2651-8 PMC7837306 32848246

[6] Martin-Gayo E Buzon MJ Ouyang Z Hickman T Cronin J Pimenova D. Potent cell-intrinsic immune responses in dendritic cells facilitate HIV-1-Specific T cell immunity in HIV-1 elite controllers. PloS Pathog (2015) 11(6):e1004930. doi: 10.1371/journal.ppat.1004930 26067651 PMC4466270

[7] Martin-Gayo E Cole MB Kolb KE Ouyang Z Cronin J Kazer SW. A reproducibility-based computational framework identifies an inducible, enhanced antiviral state in dendritic cells from HIV-1 elite controllers. Genome Biol (2018) 19(1):10. doi: 10.1186/s13059-017-1385-x 29378643 PMC5789701

[8] Al Hamrashdi M Brady G. Regulation of IRF3 activation in human antiviral signaling pathways. Biochem Pharmacol (2022) 200:115026. doi: 10.1016/j.bcp.2022.115026 35367198

[9] Mosallanejad K Kagan JC. Control of innate immunity by the cGAS-STING pathway. Immunol Cell Biol (2022) 100(6):409–23. doi: 10.1111/imcb.12555 PMC9250635 35485309

[10] Kato K Omura H Ishitani R Nureki O. Cyclic GMP-AMP as an endogenous second messenger in innate immune signaling by cytosolic DNA. Annu Rev Biochem (2017) 86:541–66. doi: 10.1146/annurev-biochem-061516-044813 28399655

[11] Wu SF Xia L Shi XD Dai YJ Zhang WN Zhao JM. RIG-I regulates myeloid differentiation by promoting TRIM25-mediated ISGylation. Proc Natl Acad Sci USA (2020) 117(25):14395–404. doi: 10.1073/pnas.1918596117 PMC7322067 32513696

[12] Zhou G Soufan O Ewald J Hancock REW Basu N Xia J. NetworkAnalyst 3.0: a visual analytics platform for comprehensive gene expression profiling and meta-analysis. Nucleic Acids Res (2019) 47(W1):W234–41. doi: 10.1093/nar/gkz240 PMC6602507 30931480

[13] Chiu YH Macmillan JB Chen ZJ. RNA Polymerase III detects cytosolic DNA and induces type I interferons through the RIG-I pathway. Cell (2009) 138(3):576–91. doi: 10.1016/j.cell.2009.06.015 PMC2747301 19631370

[14] Martin-Gayo E Gao C Chen HR Ouyang Z Kim D Kolb KE. Immunological fingerprints of controllers developing neutralizing HIV-1 antibodies. Cell Rep (2020) 30(4):984–96.e4. doi: 10.1016/j.celrep.2019.12.087 31995767 PMC6990401

[15] Vigneault F Woods M Buzon MJ Li C Pereyra F Crosby SD. Transcriptional profiling of CD4 T cells identifies distinct subgroups of HIV-1 elite controllers. J Virol (2011) 85(6):3015–9. doi: 10.1128/JVI.01846-10 PMC3067917 21177805

[16] Lahaye X Gentili M Silvin A Conrad C Picard L Jouve M. NONO detects the nuclear HIV capsid to promote cGAS-mediated innate immune activation. Cell (2018) 175(2):488–501.e22. doi: 10.1016/j.cell.2018.08.062 30270045

[17] Lahaye X Satoh T Gentili M Cerboni S Conrad C Hurbain I. The capsids of HIV-1 and HIV-2 determine immune detection of the viral cDNA by the innate sensor cGAS in dendritic cells. Immunity (2013) 39(6):1132–42. doi: 10.1016/j.immuni.2013.11.002 24269171

[18] Towers GJ Hatziioannou T Cowan S Goff SP Luban J Bieniasz PD. Cyclophilin a modulates the sensitivity of HIV-1 to host restriction factors. Nat Med (2003) 9(9):1138–43. doi: 10.1038/nm910 12897779

[19] Posch W Bermejo-Jambrina M Steger M Witting C Diem G Hörtnagl P. Complement potentiates immune sensing of HIV-1 and early type I interferon responses. mBio (2021) 12(5):e0240821. doi: 10.1128/mBio.02408-21 34634939 PMC8510548

[20] Gao D Wu J Wu YT Du F Aroh C Yan N. Cyclic GMP-AMP synthase is an innate immune sensor of HIV and other retroviruses. Science (2013) 341(6148):903–6. doi: 10.1126/science.1240933 PMC3860819 23929945

[21] Yang Z Greene WC. A new activity for SAMHD1 in HIV restriction. Nat Med (2014) 20(8):808–9. doi: 10.1038/nm.3657 25100520

[22] Li P Kaiser P Lampiris HW Kim P Yukl SA Havlir DV. Stimulating the RIG-I pathway to kill cells in the latent HIV reservoir following viral reactivation. Nat Med (2016) 22(7):807–11. doi: 10.1038/nm.4124 PMC5004598 27294875

[23] Britto AM Amoedo ND Pezzuto P Afonso AO Martínez AM Silveira J. Expression levels of the innate response gene RIG-I and its regulators RNF125 and TRIM25 in HIV-1-infected adult and pediatric individuals. Aids (2013) 27(12):1879–85. doi: 10.1097/QAD.0b013e328361cfbf 24131985

[24] Gupta S Termini JM Issac B Guirado E Stone GW. Constitutively active MAVS inhibits HIV-1 replication *via* type I interferon secretion and induction of HIV-1 restriction factors. PloS One (2016) 11(2):e0148929. doi: 10.1371/journal.pone.0148929 26849062 PMC4743994

[25] Berg RK Melchjorsen J Rintahaka J Diget E Søby S Horan KA. Genomic HIV RNA induces innate immune responses through RIG-i-dependent sensing of secondary-structured RNA. PloS One (2012) 7(1):e29291. doi: 10.1371/journal.pone.0029291 22235281 PMC3250430

[26] Wu X Yang J Na T Zhang K Davidoff AM Yuan BZ. RIG-I and IL-6 are negative-feedback regulators of STING induced by double-stranded DNA. PloS One (2017) 12(8):e0182961. doi: 10.1371/journal.pone.0182961 28806404 PMC5555650

[27] Jiang Z Wei F Zhang Y Wang T Gao W Yu S. IFI16 directly senses viral RNA and enhances RIG-I transcription and activation to restrict influenza virus infection. Nat Microbiol (2021) 6(7):932–45. doi: 10.1038/s41564-021-00907-x 33986530

[28] Koo CX Kobiyama K Shen YJ LeBert N Ahmad S Khatoo M. RNA Polymerase III regulates cytosolic RNA:DNA hybrids and intracellular microRNA expression. J Biol Chem (2015) 290(12):7463–73. doi: 10.1074/jbc.M115.636365 PMC4367256 25623070

[29] Feng H Zhang YB Gui JF Lemon SM Yamane D. Interferon regulatory factor 1 (IRF1) and anti-pathogen innate immune responses. PloS Pathog (2021) 17(1):e1009220. doi: 10.1371/journal.ppat.1009220 33476326 PMC7819612

[30] Zhou H Tang YD Zheng C. Revisiting IRF1-mediated antiviral innate immunity. Cytokine Growth factor Rev (2022) 64:1–6. doi: 10.1016/j.cytogfr.2022.01.004 35090813

[31] Harman AN Lai J Turville S Samarajiwa S Gray L Marsden V. HIV Infection of dendritic cells subverts the IFN induction pathway *via* IRF-1 and inhibits type 1 IFN production. Blood (2011) 118(2):298–308. doi: 10.1182/blood-2010-07-297721 21411754 PMC4123420

[32] Nasr N Maddocks S Turville SG Harman AN Woolger N Helbig KJ. HIV-1 infection of human macrophages directly induces viperin which inhibits viral production. Blood (2012) 120(4):778–88. doi: 10.1182/blood-2012-01-407395 22677126

[33] Calvet-Mirabent M Claiborne DT Deruaz M Tanno S Serra C Delgado-Arévalo C. Poly I:C and STING agonist-primed DC increase lymphoid tissue polyfunctional HIV-1-specific CD8(+) T cells and limit CD4(+) T-cell loss in BLT mice. Eur J Immunol (2022) 52(3):447–61. doi: 10.1002/eji.202149502 34935145

[34] Bashirova AA Martin-Gayo E Jones DC Qi Y Apps R Gao X. LILRB2 interaction with HLA class I correlates with control of HIV-1 infection. PloS Genet (2014) 10(3):e1004196. doi: 10.1371/journal.pgen.1004196 24603468 PMC3945438

[35] Sperinde JJ Choi SJ Szoka FC Jr. Phage display selection of a peptide DNase II inhibitor that enhances gene delivery. J Gene Med (2001) 3(2):101–8. doi: 10.1002/jgm.165 11318108

[36] Ahmed CM Dabelic R Bedoya SK Larkin J 3rd Johnson HM. A SOCS1/3 antagonist peptide protects mice against lethal infection with influenza a virus. Front Immunol (2015) 6:574. doi: 10.3389/fimmu.2015.00574 26617608 PMC4641302

[37] Trombetta JJ Gennert D Lu D Satija R Shalek AK Regev A. Preparation of single-cell RNA-seq libraries for next generation sequencing. Curr Protoc Mol Biol (2014) 107:4.22.1–4 17. doi: 10.1002/0471142727.mb0422s107 PMC4338574 24984854

[38] Langmead B Salzberg SL. Fast gapped-read alignment with bowtie 2. Nat Methods (2012) 9(4):357–9. doi: 10.1038/nmeth.1923 PMC3322381 22388286

[39] Li B Dewey CN. RSEM: Accurate transcript quantification from RNA-seq data with or without a reference genome. BMC Bioinf (2011) 12:323. doi: 10.1186/1471-2105-12-323 PMC3163565 21816040

