# Supplementary figures and images for: Cooperation between cGAS and RIG-I sensing pathways enables improved innate recognition of HIV-1 by myeloid dendritic cells in elite controllers
Source: Front Immunol. 2022 Dec 7;13:1017164. doi: 10.3389/fimmu.2022.1017164 (PMC9768436; doi:10.3389/fimmu.2022.1017164)

Supplemental Figure 1

A

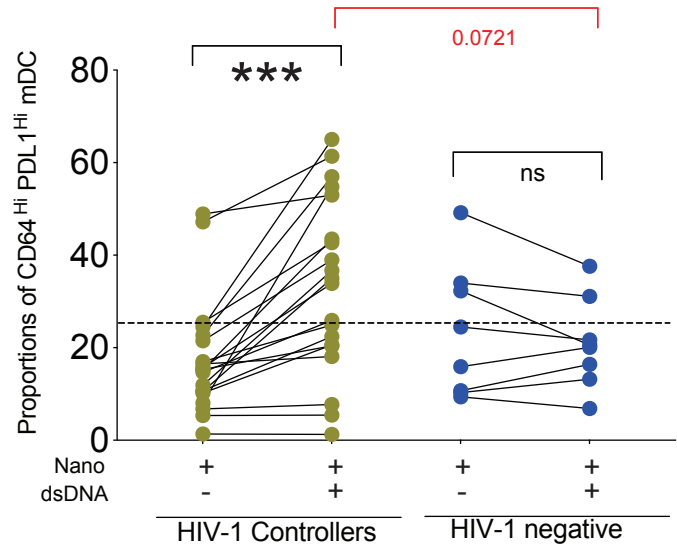

B

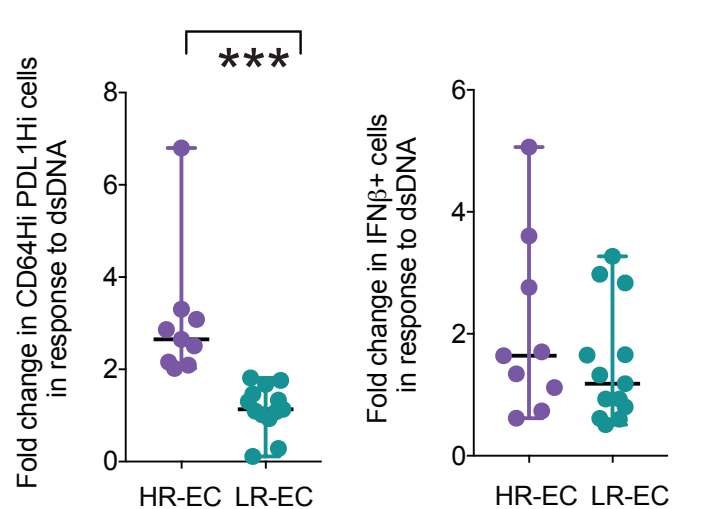

C

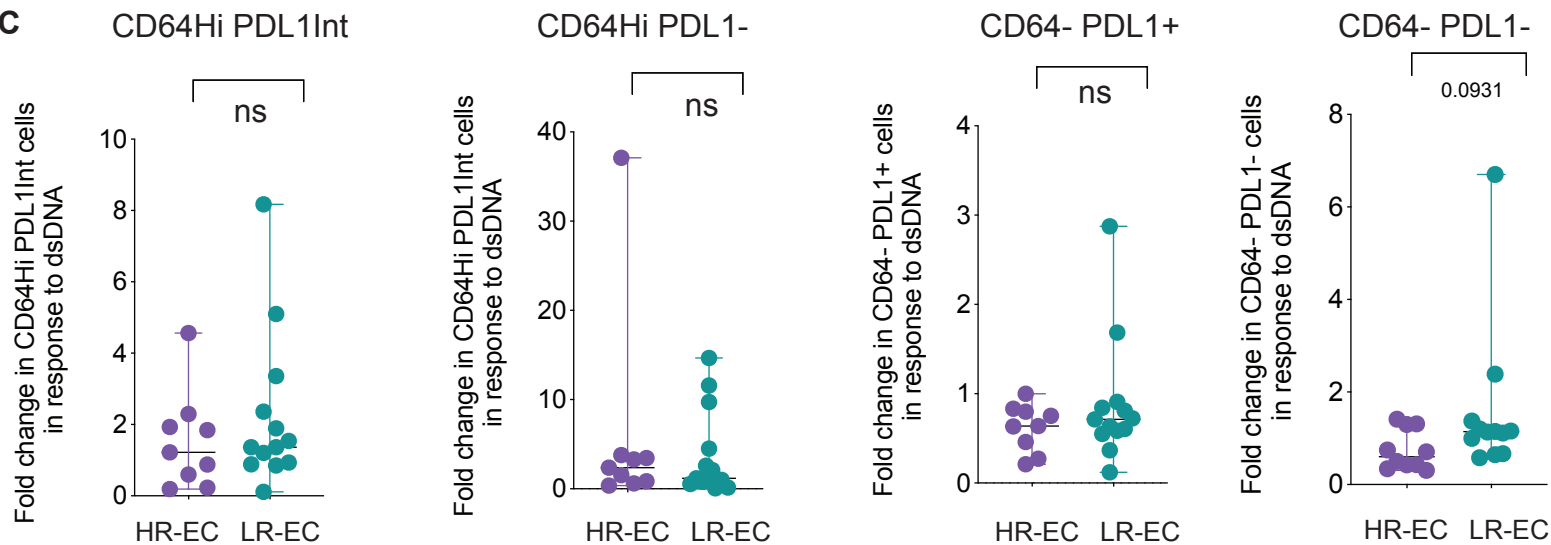

D

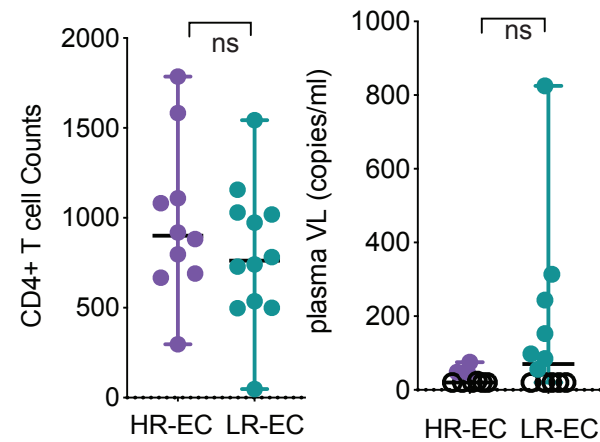

E

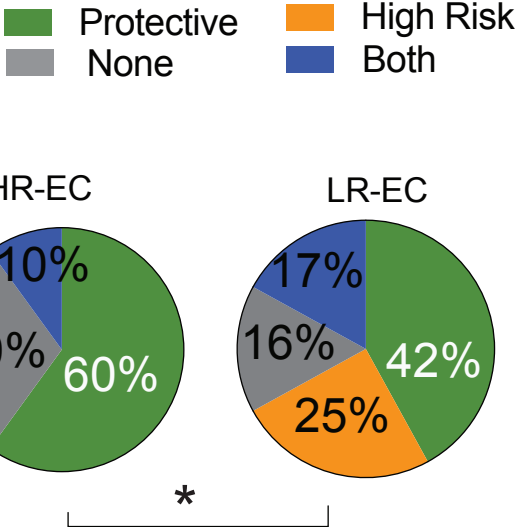

F

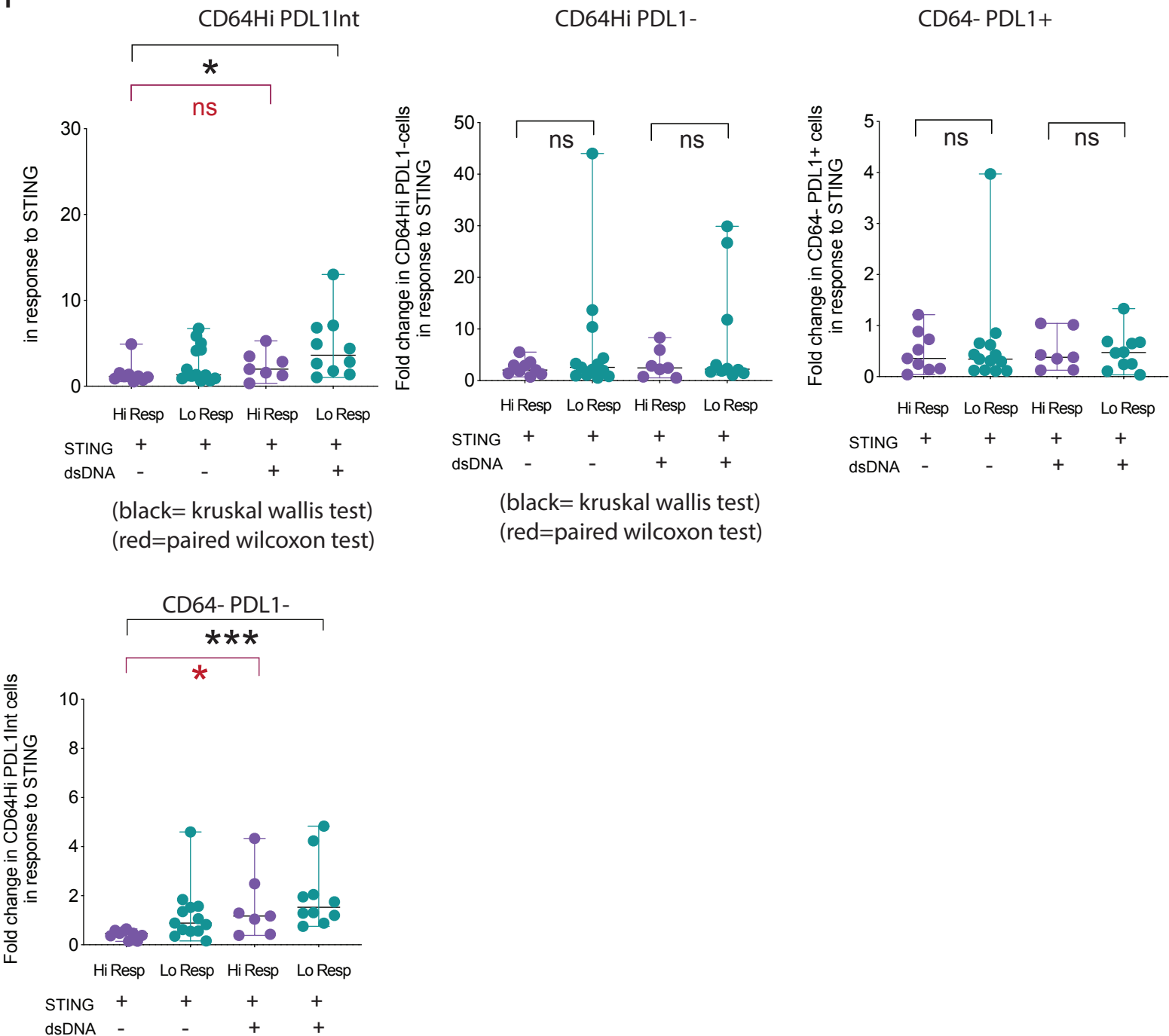

Supplement: Supplementary file 3 [file DataSheet_3.pdf]

Supplemental Figure 2

A

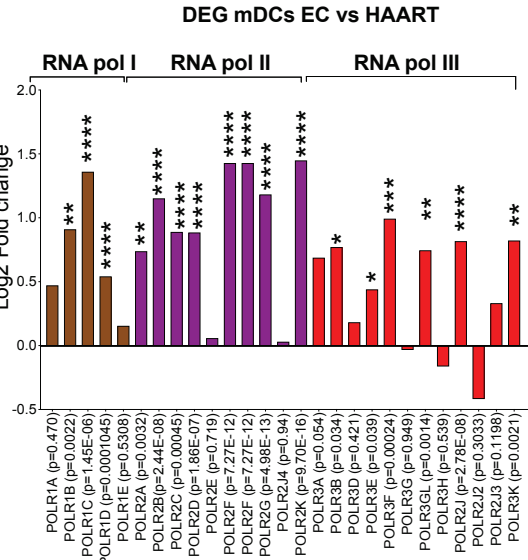

B

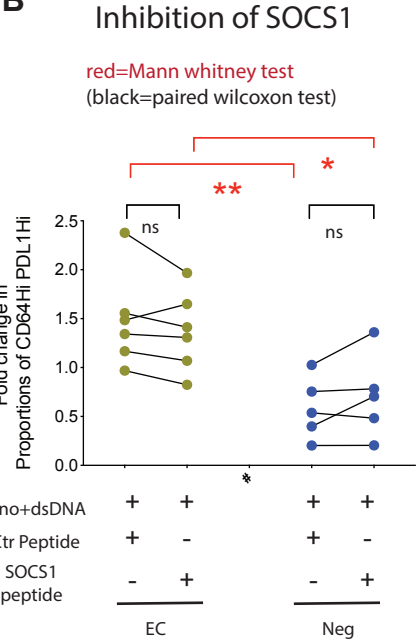

C

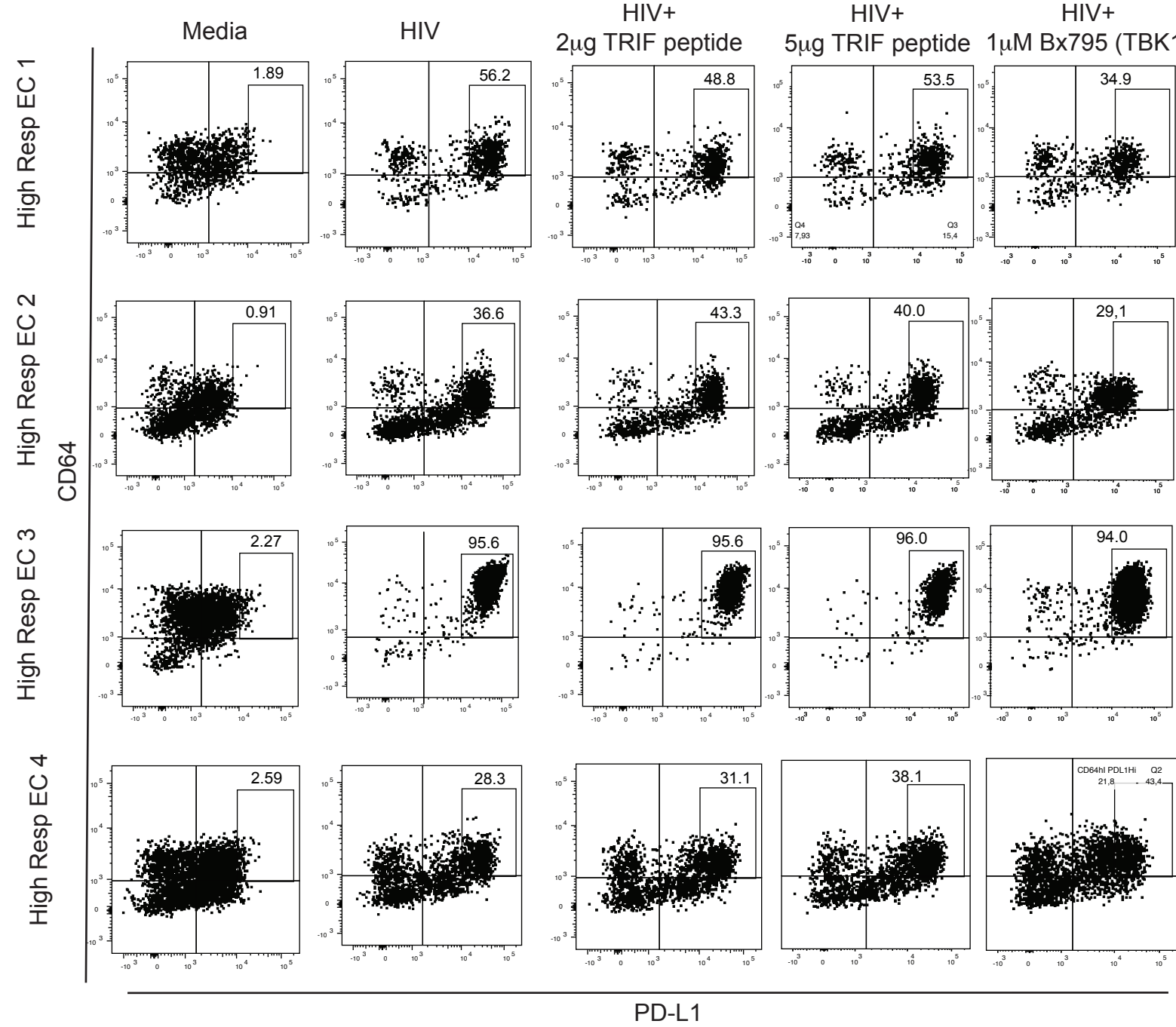

D

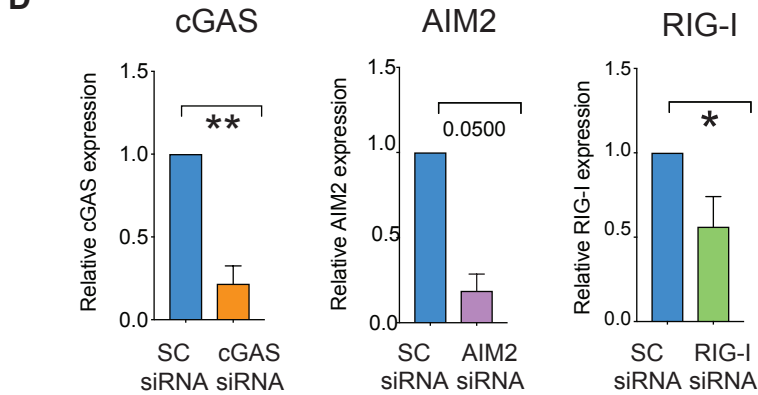

Supplement: Supplementary file 4 [file DataSheet_4.pdf]
